# Link Between Increased Satiety Gut Hormones and Reduced Food Reward After Gastric Bypass Surgery for Obesity

**DOI:** 10.1210/jc.2015-2665

**Published:** 2015-11-18

**Authors:** Anthony P. Goldstone, Alexander D. Miras, Samantha Scholtz, Sabrina Jackson, Karl J. Neff, Luc Pénicaud, Justin Geoghegan, Navpreet Chhina, Giuliana Durighel, Jimmy D. Bell, Sophie Meillon, Carel W. le Roux

**Affiliations:** Metabolic and Molecular Imaging Group (A.P.G., A.D.M., S.S., N.C., J.D.B.), Robert Steiner MRI Unit (G.D.), Medical Research Council Clinical Sciences Centre, and Centre for Neuropsychopharmacology (A.P.G.) and Computational, Cognitive, and Clinical Neuroimaging Laboratory (A.P.G., N.C.), Division of Brain Sciences, and Division of Diabetes, Endocrinology, and Metabolism (A.D.M., C.W.l.R.), Imperial College London, Hammersmith Hospital, London W12 0NN, United Kingdom; Diabetes Complications Research Centre (S.J., K.J.N., J.G., S.M., C.W.l.R.), Conway Institute, School of Medicine and Medical Science, University College Dublin, Belfield, Dublin 4, Ireland; Centre National de la Recherche Scientifique Unité Mixte de Recherche 6265 (L.P., S.M.), Centre des Sciences du Goût et de l'Alimentation, and Institut National de la Recherche Agronomique Unité Mixte de Recherche 1324 (L.P., S.M.), Centre des Sciences du Goût et de l'Alimentation, and Unité Mixte de Recherche Centre des Sciences du Goût et de l'Alimentation (L.P., S.M.), Université de Bourgogne, F-21000, Dijon, France; Research Centre for Optimal Health (J.D.B.), University of Westminster, London W1W 6UW, United Kingdom; and Department of Gastro Surgical Research and Education (C.W.l.R.), University of Gothenburg, 41345 Gothenburg, Sweden

## Abstract

**Context:**

Roux-en-Y gastric bypass (RYGB) surgery is an effective long-term intervention for weight loss maintenance, reducing appetite, and also food reward, via unclear mechanisms.

**Objective:**

To investigate the role of elevated satiety gut hormones after RYGB, we examined food hedonic-reward responses after their acute post-prandial suppression.

**Design:**

These were randomized, placebo-controlled, double-blind, crossover experimental medicine studies.

**Patients:**

Two groups, more than 5 months after RYGB for obesity (n = 7–11), compared with nonobese controls (n = 10), or patients after gastric banding (BAND) surgery (n = 9) participated in the studies.

**Intervention:**

Studies were performed after acute administration of the somatostatin analog octreotide or saline. In one study, patients after RYGB, and nonobese controls, performed a behavioral progressive ratio task for chocolate sweets. In another study, patients after RYGB, and controls after BAND surgery, performed a functional magnetic resonance imaging food picture evaluation task.

**Main Outcome Measures:**

Octreotide increased both appetitive food reward (breakpoint) in the progressive ratio task (n = 9), and food appeal (n = 9) and reward system blood oxygen level-dependent signal (n = 7) in the functional magnetic resonance imaging task, in the RYGB group, but not in the control groups.

**Results:**

Octreotide suppressed postprandial plasma peptide YY, glucagon-like peptide-1, and fibroblast growth factor-19 after RYGB. The reduction in plasma peptide YY with octreotide positively correlated with the increase in brain reward system blood oxygen level-dependent signal in RYGB/BAND subjects, with a similar trend for glucagon-like peptide-1.

**Conclusions:**

Enhanced satiety gut hormone responses after RYGB may be a causative mechanism by which anatomical alterations of the gut in obesity surgery modify behavioral and brain reward responses to food.

Roux-en-Y gastric bypass (RYGB) surgery is an effective long-term intervention for weight loss maintenance in obesity (1). Whereas RYGB and other bariatric surgeries reduce appetite, patients after RYGB also have reduced food reward responses (2–4). A reduction in food preference or appetitive behaviors for energy-dense sweet/fatty foods is reported after RYGB but not laparoscopic adjustable gastric banding (BAND), in which an inflatable band is put around the proximal stomach, or the similar vertical banded gastroplasty (2, 4–6). Appetitive reward for chocolate sweets but not vegetables is reduced after RYGB as measured by the effort expended to obtain food during a progressive ratio task (PRT) (7). Behaviors seen in rodent RYGB models are consistent with such clinical findings (2, 8).

The exact mechanisms behind these beneficial changes in food reward remain unclear because RYGB combines several manipulations, including the following: 1) reduced gastric pouch size, 2) gastric vagus nerve manipulation, 3) exclusion of food from the stomach and proximal small bowel, 4) disrupted bile flow with increased plasma bile acids, 5) altered gut microbiota, 6) aversive postprandial effects (dumping syndrome), and 7) enhanced postprandial plasma concentrations of satiety gut hormones including peptide YY (PYY), glucagon-like peptide-1 (GLP-1) and fibroblast growth factor 19 (FGF19), related to earlier delivery of food to the midgut and disrupted bile flow (3, 4, 9–12). Postprandial plasma PYY and GLP-1 concentrations are substantially higher after RYGB than BAND surgery (4, 13, 14).

We hypothesized that satiety gut hormones attenuate appetitive and anticipatory food reward responses after RYGB in humans and therefore that the acute suppression of these hormones by sc administration of the somatostatin analog octreotide would increase food reward in patients after RYGB.

In functional magnetic resonance imaging (fMRI) studies, changes in blood oxygen level-dependent (BOLD) signal reveal that activation in key brain reward areas including amygdala, caudate, nucleus accumbens, anterior insula, and orbitofrontal cortex (OFC) is lower after RYGB, when evaluating food pictures, compared with BMI-matched patients after BAND, despite both surgical groups having similarly low hunger ratings (4). This lower anticipatory fMRI reward response to food after RYGB was accompanied by lower high-energy food picture appeal ratings (4). Longitudinal reductions in high-energy food hedonics and reward responses using fMRI have also been reported after compared to before RYGB surgery (15).

Although PYY and GLP-1 additively increase satiety to reduce caloric intake (16, 17), direct evidence for their importance in the reduced food intake after RYGB remains unproven. Antagonism of GLP-1 increases food intake and body weight after RYGB in rodents but no more than in sham-operated animals (18), whereas genetic disruption of GLP-1 release or GLP-1 receptor (GLP-1R) in rodent models of bariatric surgery does not attenuate weight loss (18–20). Antagonism of PYY has not consistently increased food intake and body weight in RYGB rodent models (14, 18), but knockout of *Pyy* increased food intake and attenuated weight loss after RYGB in mice (21). Nevertheless, a broader intervention through acute suppression of several plasma gut hormones including PYY and GLP-1 secretion by administration of the somatostatin analog octreotide does increase food intake in both rats (22) and humans (23) after RYGB. Chronic sc administration of octreotide for dumping syndrome after RYGB also leads to weight gain (24).

Additional roles for these satiety gut hormones have recently been postulated for the changes in food reward after RYGB (4). PYY modulates resting activity in brain reward systems (25), and PYY and GLP-1 are additive in reducing BOLD signal in response to food pictures in the amygdala, caudate, putamen, nucleus accumbens, anterior insula, and OFC in nonobese subjects (17). Furthermore, the reduction in brain responses to food pictures with a GLP-1R agonist in nonobese and obese subjects is blocked by coadministration of a GLP-1R antagonist (26). In animal studies GLP-1R agonists reduce food intake and reward-based eating behavior, for example, reducing the effort expended to obtain sucrose in rats using a PRT, through the nucleus accumbens and mesolimbic pathways (27).

Despite these observations, no mechanistic study has yet directly established which specific anatomical or physiological perturbation after RYGB in humans reduces food reward. The objectives of this study included the following: 1) investigate the effect of satiety gut hormone suppression, using acute administration of octreotide, on food hedonic-reward responses in subjects after RYGB, using established behavioral and functional neuroimaging methodologies, and 2) compare these responses with those of subjects without enhanced postprandial gut hormone secretion, namely obese unoperated controls or patients after BAND surgery.

## Materials and Methods

### Progressive ratio task

#### Participants

Eleven patients who had previously undergone RYGB surgery were recruited from the obesity clinic at St Columcille's Hospital (Dublin, Ireland) with 10 unoperated nonobese weight-stable volunteers as age- and gender-matched controls. Inclusion criterion for the RYGB group was surgery more than 5 months previously. Exclusion criteria for both RYGB and control nonobese groups were as follows: 1) smoking, 2) pregnancy or breast-feeding, 3) significant neurological, psychiatric, or cardiovascular disease including addiction, stroke, and epilepsy other than previous depression, 4) type 1 or 2 diabetes mellitus, 5) lack of understanding of the test instructions, 6) dislike for the test stimuli, 7) active postoperative complications, and 8) current intolerance to sweet foods due to dumping syndrome.

### Progressive ratio task visit protocol

**Figure 1.**
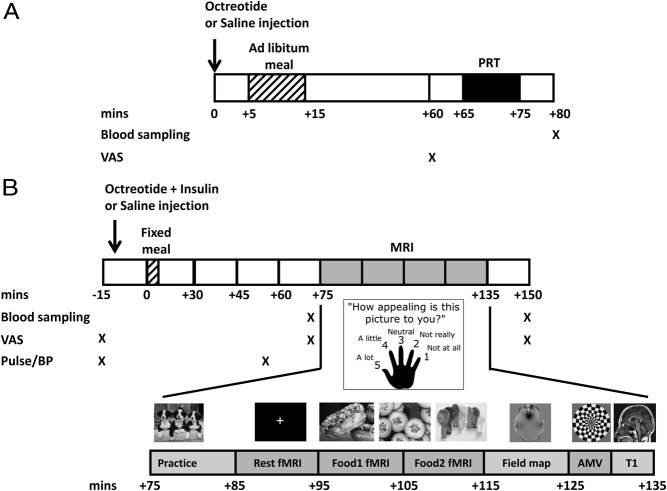
Study protocols. A, Progressive ratio task. B, Functional MRI food evaluation study. Note that the PRT lasted 10 minutes and all participants finished the task within this time period. The time interval between the end of the PRT and the +80-minute blood sample ranged from 9 to 13 minutes. AMV, auditory-motor-visual; BP, blood pressure; MRI, magnetic resonance imaging.

Participants completed two 1.5-hour sessions at the Clinical Research Centre (St Vincent University or St Columcille's Hospitals) in a within-subject, double-blind, randomized, crossover design (Supplemental Figure 1A), using sequential list randomization. After an overnight 12-hour fast, participants were injected sc with either 1 mL octreotide (Sandostatin, 100 μg; Novartis Pharmaceuticals) or 0.9% saline (23) (Figure 1A). Water was allowed during the overnight fast.

Five minutes after the injection, participants were asked to consume an ad libitum breakfast of their choice until they felt comfortably full to stimulate gut hormone release and achieve similar degrees of satiety between groups. The quantity and type of food eaten were recorded, and participants were given the exact same at the second visit, which they all consumed.

One hour after breakfast, participants rated their hunger, fullness, desire to eat, and nausea on a 10-cm visual analog scale (VAS) (4) (Supplemental Table 1). Sixty-five minutes after breakfast, participants sat in front of a computer screen with a plate of 20 chocolate sweets (M&M crispy candies, 4 kcal each, energy: 43.7% sugars, 44.1% fat; Mars UK Ltd) and performed the PRT (7). An increasing number of computer mouse clicks is needed to earn a sweet. The starting ratio to get a reward was 10 clicks with a geometric increment of 2 (ie, 10, 20, 40, etc) until the participant stopped responding, which was the breakpoint. No food or fluid was offered after the task. Participant instructions are given in the Supplemental Methods.

### Blood sampling

After the completion of the PRT, 80 minutes after breakfast, venous blood samples were collected for measurement of serum insulin, plasma glucose, total GLP-1, total PYY, and FGF19 (see Supplemental Methods).

### Functional magnetic resonance imaging study

#### Participants

Seven weight-stable patients who had previously undergone RYGB surgery and nine weight-stable patients who had undergone BAND surgery, at the Imperial Weight Centre (Charing Cross Hospital, London, United Kingdom) completed the fMRI scanning as extra visits to a previous study (see Supplemental Methods for further details) (4). Two additional new patients after RYGB completed identical study visits performing the food picture evaluation task outside the scanner because of contraindications to MR scanning. Appeal ratings were therefore available for nine patients after RYGB, and fMRI scans for seven patients after RYGB and nine patients after BAND surgery. Exclusion/inclusion criteria were as described (4).

### Scanning visit protocol

After an overnight fast (water was allowed), patients were randomized, in a double-blind, crossover, within-subject design (Figure 1B), to receive, 10 minutes before breakfast, either two sc injections of 0.9% saline or octreotide (Sandostatin, 100 μg; Novartis Pharmaceuticals) (23) plus a short-acting insulin to attenuate postprandial hyperglycemia from the suppression of insulin (Actrapid, 0.075–0.10 U/kg; Novo Nordisk) as two separate injections. Subjects then received a standardized milkshake breakfast (one sachet Complan plus 200 mL whole milk, giving 385 kcal; energy: carbohydrate 45.6%, fat 16.0%, protein 38.4%) at t = 0 minutes. Participants were monitored for a total of 165 minutes to ensure they did not become hypoglycemic.

### Food picture evaluation fMRI paradigm

During the fMRI paradigm, starting at +95 minutes, four types of color photographs were presented in a block design split across two runs: 1) 60 high-energy foods (eg, pizza, cakes, and chocolate); 2) 60 low-energy foods (eg, salads, vegetables, fish); 3) 60 nonfood-related household objects (eg, furniture, clothing), with each category in 10 blocks; and 4) 180 Gaussian blurred images of the other pictures, as described (4, 28).

While each image was on display to subjects in the scanner, they were asked to immediately and simultaneously rate how appealing each picture was to them using a five-button handheld keypad (1, not at all; 5, a lot) (4, 28).

### Auditory-motor-visual control fMRI paradigm

In the 6-minute auditory-motor-visual (AMV) control task, over nine blocks, subjects performed two of the following tasks simultaneously: 1) listening to a story, 2) tapping their right index finger once every second, or 3) watching a 4-Hz color (yellow/blue) flashing checkerboard, with each task performed in six blocks (4, 28). This control fMRI task was used to ensure that changes in the BOLD signal were not nonspecific, eg, due to alterations in neurovascular coupling.

### fMRI analysis

Preprocessing used the fMRI Expert Analysis Tool version 5.98 software, part of the FMRIB Software Library, version 4.1 (www.fmrib.ox.ac.uk/fsl), as described (4, 28).

The average (median) BOLD signal for the contrast high-energy food greater than object and low-energy food greater than object were calculated for each subject at each visit in these functional regions of interest (fROIs): amygdala, nucleus accumbens, anterior insula, and caudate nucleus (4, 28). The fROIs were determined from a separate cohort of 24 overweight/obese subjects who underwent an identical protocol after fasting overnight, without administration of octreotide/Insulin, for the food (high energy or low energy) greater than object contrast at voxel-wise false discovery rate corrected *P* < .05, as previously described (4) (and see Supplemental Methods). Excess signal dropout precluded the use of an OFC fROI. Similar functional localizers were made for the control AMV task for bilateral superior posterior temporal gyrus (auditory), left precentral gyrus (motor), and bilateral lingual gyrus (visual) from the same separate cohort of 24 overweight/obese subjects, as described (4).

### Appetite ratings

VAS ratings (0–10 cm) of appetite and other symptoms were recorded at serial time points to measure hunger, pleasantness to eat, volume of food wanting to eat, fullness, and sickness (4, 28).

### Blood sampling

The concentration of serum insulin, plasma glucose, insulin and total GLP-1, total PYY, and FGF19 (see Supplemental Methods for assay methods) over the scanning period was calculated using the mean of concentrations at time point +70 minutes (just before the fMRI scan) and time point +150 minutes (just after the fMRI scan).

### Statistical analysis

SPSS version 21 (IBM) and Prism version 5 (GraphPad Software Inc) were used to perform the statistical analyses. Results are presented as mean ± SEM or median [interquartile range] for data that was not normally distributed (Kolmogorov-Smirnov test) and range. Significance was taken as *P* < .05. Effect sizes are described as mean difference ± SEM, 95% confidence intervals (CI), and Cohen's d values.

Between-group comparisons used an unpaired Student's *t* test, Mann-Whitney *U* test (if data not normally distributed), or Fisher's exact test for categorical variables to examine group differences. Within-group comparisons used a paired *t* test or Wilcoxon rank sum test (if data were not normally distributed) to examine the response to treatment (octreotide vs saline) separately in each group. In the fMRI study, a two-way, repeated-measures ANOVA including treatment (octreotide vs saline) and food picture energy density (high energy vs low energy food) as within-subject factors was used to determine the effects of treatment, energy density, and treatment × energy density interaction, separately within each group, with post hoc Fisher's least significance difference (LSD) test. A multivariate analysis was performed using all fROIs as well as a univariate analysis for each individual fROI or an average signal across all fROIs.

To directly compare the effects of octreotide between groups, univariate, two-way, repeated-measures ANOVAs were performed including treatment (octreotide vs saline) as within-subject factor and group as between-subject factor to determine effects of treatment, group, and treatment × group interaction, with a post hoc Fisher's LSD test. In the fMRI analyses, energy density (high energy vs low energy food) was also included as an additional within-subject factor in a univariate, three-way, repeated-measures ANOVA. Given our a priori hypothesis of enhanced effects of octreotide in the RYGB groups, we illustrate the post hoc effects of treatment within each group, irrespective of the treatment × group interaction effect.

Pearson correlation coefficients r were determined from linear regression analysis to examine the relationship between the change in outcome variables between octreotide and saline visits and either the variable at the saline visit or the change in plasma gut hormones between the octreotide and saline visits.

## Results

### 

#### Progressive ratio task

The healthy nonobese control group and adults after RYGB were of similar age, gender ratio, and ethnicity, but the RYGB group remained significantly heavier than controls (Table 1). Five of the 10 controls and five of the 11 RYGB subjects had the saline injection at the first visit.

**Table 1.** Participant Characteristics in Progressive Ratio Task and fMRI Food Evaluation Studies

	Progressive Ratio Task Study			fMRI Food Evaluation Study^a^			
	Controls	RYGB	*P* Value^b^	RYGB (All)	RYGB (Scanned)^c^	BAND	*P* Value^d^
n	10	11		9	7	9	
Age, y	45.3 ± 3.4 (31.0, 62.0)	50.5 ± 9.2 (34.0, 68.0)	.25	47.0 ± 1.8 (42.0, 59.0)	46.0 ± 1.0 (42.0, 50.0)	41.8 ± 3.8 (26.0, 59.0)	.22
Gender (male/female)	6/4	6/5	1.00	3/6	2/5	1/8	.58
European Caucasians, n, %	10 (100%)	11 (100%)	1.00	9 (100%)	7 (100%)	6 (75%)	.21
Preoperative BMI, kg/m^2^	n/a	54.0 ± 2.8 (41.2, 71.0)	n/a	52.8 ± 2.0 (38.3, 74.6)	55.2 ± 5.3 (38.3, 74.6)	51.7 ± 4.8 (36.5, 86.2)	.84
Current BMI, kg/m^2^	24.5 ± 0.6 (20.6, 26.9)	38.8 ± 2.0 (25.8, 49.1)	**.02**	37.3 ± 2.6 (29.4, 48.8)	38.6 ± 3.1 (29.4, 48.8)	33.2 ± 6.3 (25.2, 43.8)	.25
Current weight, kg	71.8 ± 3.2 (56.1, 88.20)	110.6 ± 8.9 (63.5, 169.9)	**<.001**	105.6 ± 6.2 (75.0, 129.3)	109.0 ± 6.4 (82.2, 129.3)	91.3 ± 4.8 (75.5, 115.1)	.10
Current body fat, %	ND	ND	n/a	42.0 ± 3.9 (23.1, 55.1)	41.5 ± 5.0 (23.1, 55.1)	40.2 ± 3.2 (23.6, 52.0)	.73
Weight loss, % preoperative weight	n/a	27.2 ± 3.9 (3.0, 47.0)	n/a	28.3 ± 2.0 (21.1, 38.3)	29.1 ± 2.4 (21.1, 38.3)	27.4 ± 4.0 (10.0, 52.0)	.84
Time since surgery, mo	n/a	28.4 ± 5.5 (5.6, 68.0)	n/a	15.6 ± 2.5 (5.2, 23.9)	14.2 ± 3.0 (5.2, 23.9)	15.3 ± 3.6 (4.0, 36.0)	.95
Preoperative DM, n, %	n/a	2 (18%)	n/a	5 (56%)	4 (57%)	0 (0%)	**.03**
Current DM, n, %	0 (0%)	1 (9%)	1.00	1 (11%)	1 (14%)	0 (0%)	1.00
Preoperative BED, n, %	n/a	ND	n/a	2 (22%)	1 (14%)	2 (22%)	1.00
Current BED, n, %	ND	ND	n/a	1 (11%)	1 (14%)	0 (0%)	1.00

Abbreviations: BED, binge eating disorder; BMI, body mass index; DM, type 2 diabetes mellitus; n/a, not applicable; ND, not determined. Data are presented as mean ± SEM and (minimum, maximum), or n (percentage). Bold text indicates *P* < .05.

a Data are calculated from the average of the two visits in the fMRI evaluation study.

b *P* value for comparison of controls vs RYGB, using independent two-tailed *t* test for continuous variables or Fisher's exact test for prevalence data.

c Excluding two participants with contraindications to MRI.

d *P* value for comparison of RYGB (all) vs BAND, using independent two-tailed *t* test for continuous variables or Fisher's exact test for prevalence data.

### Glucose and hormone responses

**Figure 2.**
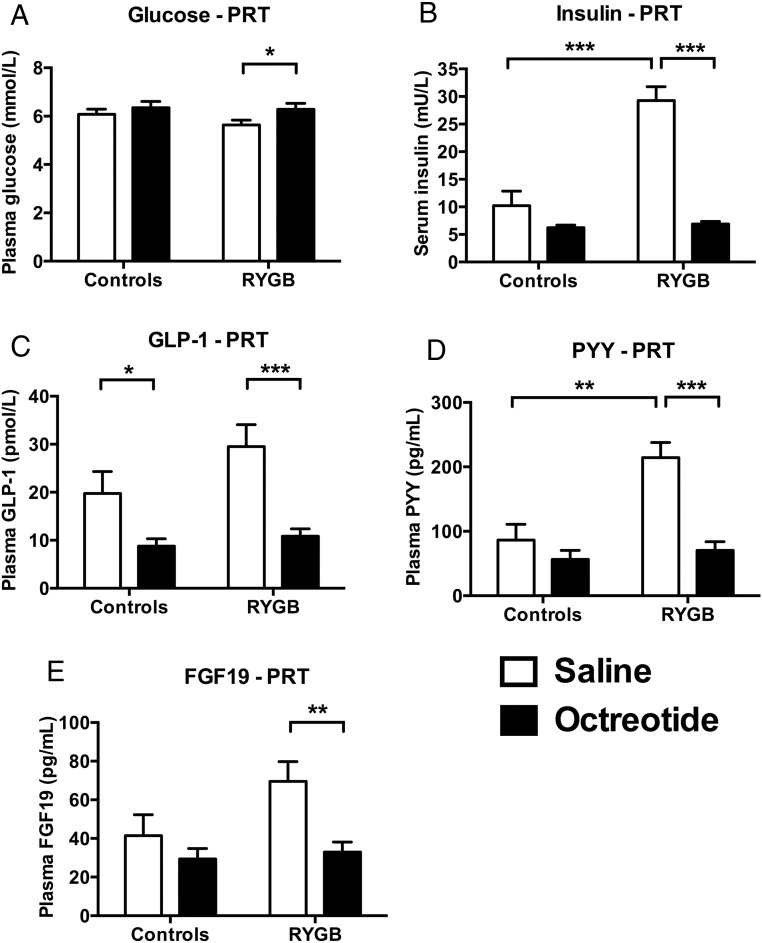
Octreotide suppressed postprandial plasma gut hormones in progressive ratio task. A, Plasma glucose. Octreotide significantly increased plasma glucose in patients after RYGB (mean difference [95% CI] 0.6 [0.2, 1.1] mmol/L, F[1, 19] = 7.84, *P* = .011, Cohen's d = 0.84) but not in controls (0.3 [−0.2, 0.8] mmol/L, F[1, 19] = 1.25, *P* = .28, Cohen's d = 0.00). To convert millimoles per liter to milligrams per deciliter, multiply by 18.01. B, Serum insulin. Octreotide significantly decreased serum insulin in patients after RYGB (mean difference [95% CI] −22.3 [−27.7, −16.9] mU/L, F[1, 19] = 74.88, *P* < .001, Cohen's d = 2.05) but not in controls (−4.0 [−9.6, 1.7] mU/L, F[1, 19] = 2.15, *P* = .10, Cohen's d = 1.08). To convert milliunits per liter to picomoles per liter, multiply by 7.175. C, Plasma GLP-1. Octreotide significantly decreased plasma total GLP-1 both in patients after RYGB (mean difference [95% CI] −18.7 [−27.7, −9.6] pmol/L, F[1, 18] = 18.81, *P* < .001, Cohen's d = 0.98) and in controls (−11.0 [−20.0, −1.93] pmol/L, F[1, 8] = 6.50, *P* = .020, Cohen's d = 1.49). D, Plasma PYY. Octreotide significantly decreased plasma total PYY in patients after RYGB (mean difference [95% CI] −143.8 [−193.3, −94.4] pg/mL, F[1, 19] = 37.08, *P* < .001, Cohen's d = 1.48) but not in controls (−30.0 [−81.8, 21.9] pg/mL, F[1, 19] = 1.46, *P* = .24, Cohen's d = 0.62). To convert picograms per milliliter to picomoles per liter, multiply by 0.25. E, Plasma FGF19. Octreotide significantly decreased plasma FGF19 in patients after RYGB (mean difference [95% CI] −36.6 [−59.4, −13.7] pg/mL, F[1, 19] = 11.23, *P* = .003, Cohen's d = 0.91) but not in controls (−12.1 [−36.1, 11.9] pg/mL, F[1, 19] = 1.12, *P* = .30, Cohen's d = 0.47). Data represent mean ± SEM (n = 11 per group except RYGB group, GLP-1, n = 10). White bars, saline; black bars, octreotide. *, *P* < .05, ***, *P* < .001 with post hoc Fisher's LSD test. For statistics of repeated-measures ANOVA for effects of group, treatment, and group × treatment interaction, see Supplemental Table 2.

As expected, compared with saline administration, octreotide suppressed postprandial plasma PYY and GLP-1, and serum insulin, but also plasma FGF19, concentrations in patients after RYGB (Figure 2, B–E, and Supplemental Table 2). In controls this suppressive effect of octreotide was significant only for GLP-1. However, there was a significantly greater suppression of insulin and PYY with octreotide in patients after RYGB than controls but not for GLP-1 or FGF19 (Figure 2, B and D, and Supplemental Table 2).

Octreotide significantly increased postprandial plasma glucose in patients after RYGB but not controls, although concentrations remained at 7.7 mmol/L or less (≤139 mg/dL), ie, not in the hyperglycemic range, and there was no significant difference between groups in the effect of octreotide on plasma glucose (Figure 2A and Supplemental Table 2).

At the saline visit, postprandial serum insulin and plasma PYY concentrations were greater in patients after RYGB than nonobese control subjects (insulin: mean difference RYGB minus control [95% CI] −19.0 [11.4, 26.7] mU/L, F[1, 19] = 27.41, *P* < .001, Cohen's d = 2.29; PYY: 128.0 [56.6, 199.3] pmol/L, F[1, 19] = 14.08, *P* = .001, Cohen's d = 1.64), with a similar trend for plasma FGF19 (28.0 [−3.2, 59.2] pg/mL, F[1, 19] = 3.54, *P* = .075, Cohen's d = 0.82) (Figure 2 and Supplemental Table 2). However, plasma glucose and GLP-1 concentrations were not significantly different between groups (glucose: −0.4 [−1.0, 0.2] mmol/L, F[1, 19] = 2.39, *P* = .14, Cohen's d = 0.68; GLP-1: 19.8 [−3.8, 23.3] pmol/L, F[1, 18] = 2.28, *P* = .15, Cohen's d = 0.67) (Figure 2 and Supplemental Table 2).

### Behavioral responses

**Figure 3.**
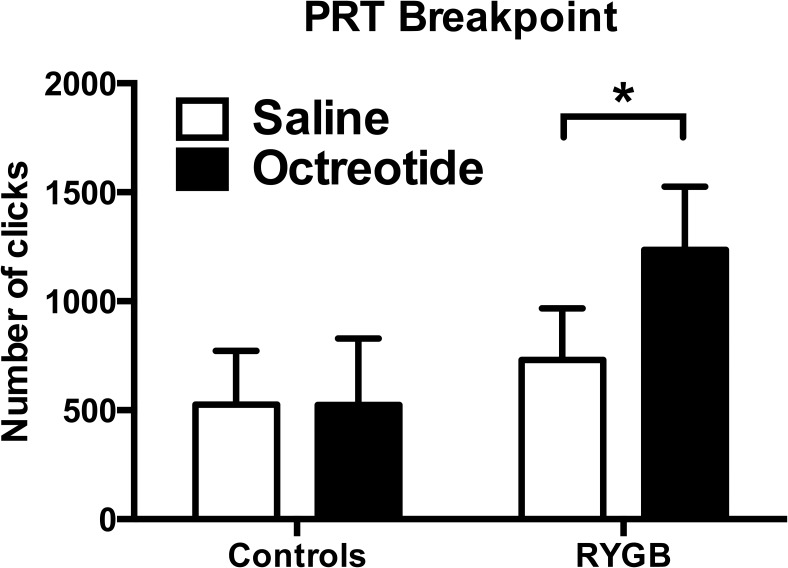
Octreotide increased food reward responses in progressive ratio task. Octreotide significantly increased the total number of clicks (breakpoint) in patients after RYGB (mean difference [95% CI] 503 [218, 788], F[1, 19] = 13.66, *P* = .002, Cohen's d = 0.62) but not in controls (effect size −1 [−300, 298], F[1, 19]=0.00, *P* = .99, Cohen's d = 0.00). Overall ANOVA: group (between subject), F(1, 19) = 1.53, *P* = .23; treatment (within subject), F(1, 19) = 6.47 *P* = .02; group × treatment interaction, F(1, 19) = 6.54, *P* = .02.

The breakpoint of the PRT, which represents the amount of work the subject did to obtain the reward, was not significantly different between RYGB and control groups after saline administration (Figure 3 and Supplemental Figure 1). There was a significantly greater effect of octreotide in the RYGB compared with the control group, with octreotide significantly increasing the breakpoint in patients after RYGB (Figure 3 and Supplemental Figure 1) but not the control group. By contrast, octreotide had no significant effects on the VAS ratings of hunger, fullness, or nausea in either group (Supplemental Table 1).

### Hormone-behavior correlations

There was no significant correlation between the increase in PRT breakpoint, and the decrease in plasma PYY, GLP-1, or FGF19, with octreotide administration (both Δoctreotide-saline) in patients after RYGB alone, or in the RYGB and control group combined (Supplemental Table 3).

#### fMRI study

Appeal ratings were available for nine RYGB subjects and fMRI scans for seven RYGB subjects. The RYGB and BAND groups were of similar age, gender ratio, ethnicity, preoperative BMI, current BMI, and percentage body fat, percentage weight loss since surgery, and time since surgery (Table 1).

### Glucose and hormone responses

**Figure 4.**
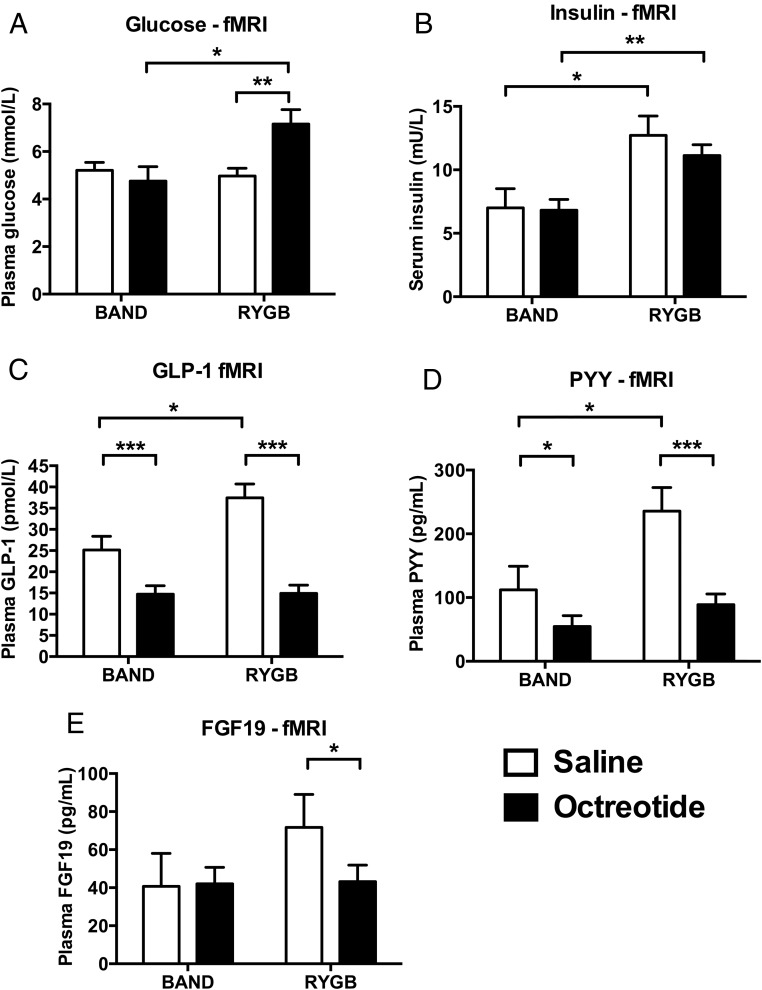
Octreotide suppressed postprandial plasma gut hormones in fMRI food evaluation study. A, Plasma glucose. Octreotide significantly increased plasma glucose in patients after RYGB (mean difference [95% CI] 2.2 [0.9, 3.5] mmol/L, F[1, 16] = 12.78, *P* = .003, Cohen's d = 2.31) but not in patients after BAND (0.5 [−0.8, 1.8] mmol/L, F[1, 16] = 0.55, *P* = .47, Cohen's d = 0.48). To convert millimoles per liter to milligrams per deciliter, multiply by 18.01. B, Serum insulin. Octreotide did not have a significant effect on serum insulin in patients after RYGB (mean difference [95% CI] −1.6 [−5.1, 1.9] mU/L, F[1, 16] = 0.94, *P* = .35, Cohen's d = 0.26) or in patients after BAND (−0.2 [−3.7, 3.3] mU/L, F[1, 16] = 0.01, *P* = .92, Cohen's d = 0.11). To convert milliunits per liter to picomoles per liter, multiply by 7.175. C, Plasma GLP-1. Octreotide significantly decreased plasma total GLP-1 in patients after RYGB (mean difference [95% CI] −22.6 [−27.3, −17.8] pmol/L, F[1, 16] = 102.16, *P* < .001, Cohen's d = 1.80), with a significantly smaller decrease in patients after BAND (−10.4 [−15.1, −5.7] pmol/L, F[1, 16] = 21.74, *P* < .001, Cohen's d = 1.90). D, Plasma PYY. Octreotide significantly decreased plasma total PYY in patients after RYGB (mean difference [95% CI] −146.8 [−203.5, −90.1] pg/mL, F[1, 16] = 30.12, *P* < .001, Cohen's d = 0.95), with a significantly smaller decrease in patients after BAND (−57.6 [−114.3, −0.88] pg/mL, F[1, 16] = 4.63, *P* = .047, Cohen's d = 1.95). To convert picograms per milliliter to picomoles liter, multiply by 0.25. E, Plasma FGF19. Octreotide significantly decreased plasma FGF19 in patients after RYGB (mean difference [95% CI] −28.5 [−50.4, −6.5] pg/mL, F[1, 16] = 7.54, *P* = .014, Cohen's d = 0.40) but not in patients after BAND (−12.1 [−36.1, 11.9] pg/mL, F[1, 16] = 0.02, *P* = .90, Cohen's d = 0.08). Data represent mean ± SEM (n = 9 per group). White bars, saline; black bars, octreotide. *, *P* < .05, ***, *P* < .001 with post hoc Fisher's LSD test. For statistics of repeated-measures ANOVA for effects of group, treatment, and interaction, see Supplemental Table 4.

At the saline visit, postprandial serum insulin and, as expected, the plasma GLP-1 and PYY concentrations were significantly greater in patients after RYGB than after BAND (insulin: mean difference RYGB minus BAND [95% CI] 5.7 [1.2, 10.3] mU/L, F[1, 16] = 7.07, *P* = .017, Cohen's d = 1.25; GLP-1: 12.3 [2.6, 22.0] pmol/L, F[1, 16] = 7.26, *P* = .016, Cohen's d = 1.27; PYY: 123.4 [12.6, 234.1] pg/mL, F[1, 16] = 5.58, *P* = .031, Cohen's d = 1.11) (Figure 4 and Supplemental Table 2). However, there was no significant difference in plasma glucose or FGF19 concentrations between the RYGB and BAND groups (glucose: −0.2 [−1.2, 0.8] mmol/L, F[1, 16] = 0.27, *P* = .61, Cohen's d = 0.25; FGF19: 31.0 [−20.9, 83.0] pg/mL, F[1, 16] = 1.60, *P* = .22, Cohen's d = 0.60) (Figure 4 and Supplemental Table 2).

As expected, the gut hormone-suppressive effects of octreotide in the RYGB group were similar in the fMRI study to that in the PRT study. Compared with the saline administration, octreotide significantly suppressed postprandial plasma GLP-1, PYY, and again FGF19 over the scanning period (Figure 4, C–E). Interestingly, octreotide also suppressed postprandial GLP-1 and PYY but not postprandial FGF19 in the BAND group (Figure 4, C–E). Octreotide had a greater suppressive effect on plasma GLP-1 and PYY in the RYGB group than the BAND group, with a similar trend seen for FGF19 (Supplemental Table 4). This appears to be a consequence of higher postprandial plasma gut hormone concentrations, particularly for GLP-1, in patients after RYGB than BAND at the saline visit.

The coadministration of exogenous insulin avoided the potential confound of significantly lower postprandial serum insulin concentrations after octreotide in both the RYGB and BAND groups (Figure 4B). In the BAND group, coadministration of octreotide and exogenous insulin did not increase postprandial glucose concentrations (Figure 4A). However, postprandial glucose concentrations still increased after octreotide in the RYGB group, although the concentrations were 9.5 mmol/L or less (<171 mg/dL) (Figure 4A and Supplemental Table 4). None of our patients reported any hypoglycemic adverse events after discharge from the clinical research facility.

### Food hedonic responses

**Figure 5.**
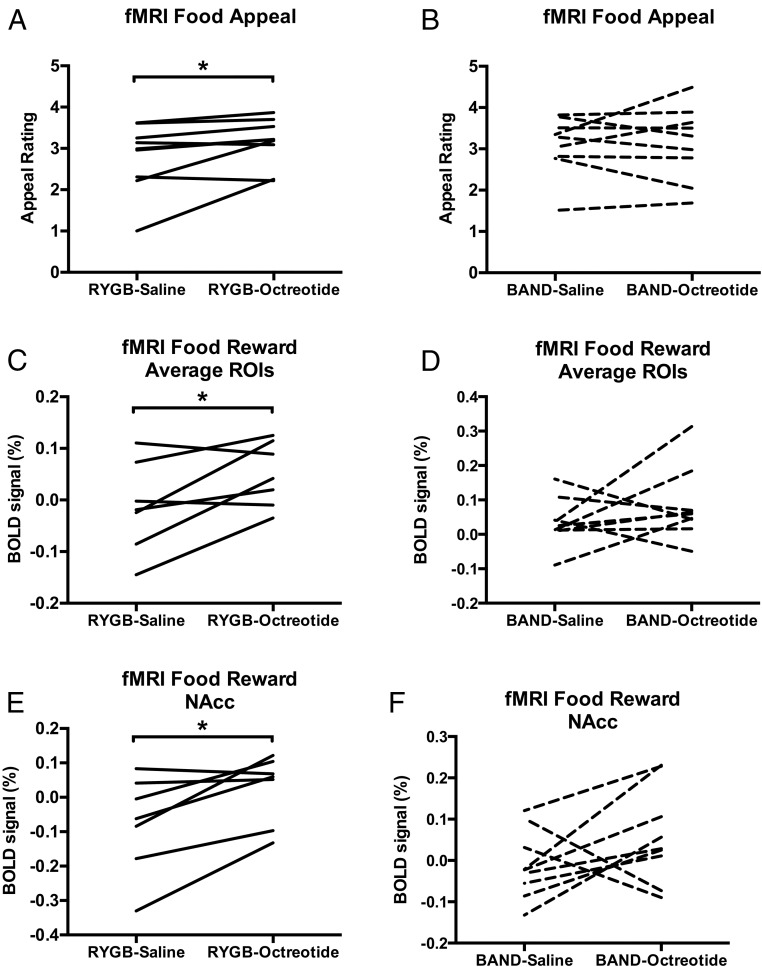
Effect of octreotide on hedonic and brain reward system responses in fMRI food evaluation task after RYGB and BAND. A and B, Octreotide increased food appeal ratings in patients after RYGB (mean difference [95% CI] 0.35 [0.01, 0.70], F[1, 8] = 5.48, *P* = .047, Cohen's d = 0.43) (A) but not BAND (mean difference [95% CI] 0.049 [−0.38, 0.48], F[1, 8] = 0.07, *P* = .80, Cohen's d = 0.07) (B). C and D, Octreotide significantly increased average BOLD signal (percentage) to any food picture averaged across all four fROIs (nucleus accumbens, caudate, anterior insula, amygdala) in patients after RYGB (mean difference [95% CI] 0.062 [0.003, 0.122], F[1, 6] = 6.54, *P* = .043, Cohen's d = 0.77) (C) but not BAND (mean difference [95% CI] 0.047 [−0.051, 0.146], F[1, 8] = 1.21, *P* = .30, Cohen's d = 0.68) (D). E and F, Octreotide significantly increased average BOLD signal (percentage) to any food picture in the nucleus accumbens alone in patients after RYGB (0.102 [0.023, 0.180], F[1, 6] = 10.11, *P* = .019, Cohen's d = 0.79) (E) but not BAND (mean difference [95% CI] 0.069 [−0.037, 0.174], F[1, 8] = 2.27, *P* = .17, Cohen's d = 0.82) (F). Analysis from repeated-measures ANOVA performed separately in each group, RYGB (n = 7, except food appeal rating, n = 9) or BAND (n = 9), with treatment (octreotide or saline) and energy density of food pictures (high or low) as within-subject factors. There was no significant effect of food picture energy density or energy density × treatment interaction for any fROI in either group. *, *P* < .05, octreotide vs saline with post hoc Fisher's LSD test. For statistics of repeated-measures ANOVA for effects of group, treatment, and interaction, see Supplemental Table 5.

Compared with saline, octreotide significantly increased the food appeal ratings in patients after RYGB (Figure 5A) but not in patients after BAND (Figure 5B). However, on direct comparison, there was no significant difference in the effect of octreotide on food appeal ratings between the surgical groups (Supplemental Figure 2A and Supplemental Table 5).

### BOLD signal responses

In the RYGB group, multivariate analysis including all the a priori fROIs (nucleus accumbens, caudate, anterior insula, amygdala) revealed that octreotide significantly increased the BOLD signal during the evaluation of food pictures (F[4, 3] = 13.86, *P* = .028), independent of the food picture energy density (energy density × treatment interaction [F[4, 3] = 0.57, *P* = .71]). In univariate analyses of individual fROIs in the RYGB group, octreotide significantly increased the BOLD signal during the food picture evaluation averaged across all the a priori fROIs and in the nucleus accumbens alone (Figure 5, C and E). Interestingly a lower food appeal and BOLD signal during food evaluation after saline was associated with a greater increase in these variables with octreotide administration (Supplemental Figure 3).

By contrast, in a multivariate analysis of all fROIs in the BAND group, octreotide had no significant effect on BOLD signal during evaluation of food pictures in the a priori fROIs: (F[4, 5] = 0.45, *P* = .77), independent of the food picture energy density (energy density × treatment interaction [F[4, 5] = 0.26, *P* = .89]). Similarly in univariate analyses of individual fROIs in the BAND group, octreotide had no significant effect on BOLD signal during food picture evaluation averaged across all the a priori ROIs or in the nucleus accumbens (Figure 5, D and F).

However, on direct comparison, there was no significant difference in the effect of octreotide on BOLD signal averaged across all four a priori fROIs, the nucleus accumbens, or other individual ROIs (Supplemental Figure 2 and Supplemental Table 5).

### Hormone-BOLD signal correlations

**Figure 6.**
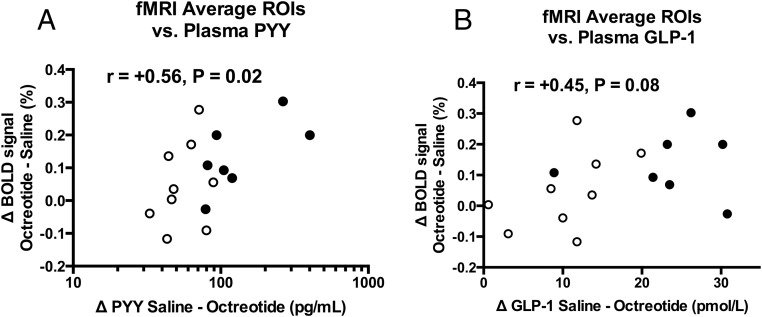
Greater suppression of plasma PYY and GLP-1 with octreotide is associated with greater increase in brain reward system response in fMRI food evaluation task after bariatric surgery. The magnitude of the increase in BOLD signal (percentage) averaged across the four fROIs (nucleus accumbens, caudate, anterior insula, amygdala) to any food picture (high energy or low energy vs objects) with octreotide (delta octreotide-saline) was positively correlated with the decrease in plasma concentration of PYY with octreotide (delta saline-octreotide) (A), with a similar trend for plasma concentrations of GLP-1 (B), independent of the surgical group. r represents Pearson correlation coefficient. Open circles represent patients after BAND (n = 9) and filled circles patients after RYGB (n = 7). Results for PYY are displayed as log_10_ scale because data were not normally distributed. One RYGB subject with a decrease in plasma PYY after octreotide of 401.3 pg/mL was an outlier (> 2 SD) in these analyses. The relationship between delta BOLD signal and delta plasma PYY remained significant when excluding this subject (r[14] = +0.54, *P* = .036).

In the combined surgical group analysis (n = 16), there was a significant positive correlation between the increase in the BOLD signal to the food pictures averaged across the a priori fROIs with octreotide administration and the decrease in plasma PYY with octreotide administration (r[15] = +0.56, *P* = .02, Figure 6A), with a similar trend for GLP-1 (r [15] = +0.45, *P* = .08, Figure 6B), but not FGF19 (r[15] = +0.06, *P* = .83).

### Confounding variables

As with the PRT study, octreotide had no significant effects in either the RYGB or BAND groups on the following: 1) VAS ratings of hunger or fullness (Supplemental Tables 6 and 7), 2) BOLD signal during the control auditory-motor-visual fMRI task (suggesting a lack of nonspecific effects), 3) motion during scanning, 4) emotional ratings (Supplemental Tables 8 and 9), or 5) postprandial signs and symptoms of the dumping syndrome (Supplemental Tables 10 and 11).

## Discussion

In two cohorts of patients after RYGB using complimentary behavioral tests and functional neuroimaging, we demonstrate that lowering postprandial plasma satiety gut hormones by acute octreotide administration increased the breakpoint for a chocolate sweet in a PRT and increased food appeal ratings and brain reward system activation during evaluation of food pictures.

These measures of food reward are of direct relevance to actual habitual eating behavior, food intake, and changes in body weight, although these are not examined in the current study. The magnitude of change in the breakpoint for chocolate sweets in the PRT have been correlated with the degree of weight loss after RYGB (7), and the breakpoint for different foods correlate with habitual dietary intake and energy intake in test meals (29). The BOLD signal in brain reward regions, including the nucleus accumbens, caudate, amygdala, and insula, to anticipatory food cues correlate with pleasantness ratings of ingested food after bariatric surgery (4), and predict food intake at test meals (30, 31) and weight loss during lifestyle modification (32). The caudate and nucleus accumbens are involved in habitual and goal- and reward-directed behavior. The anterior insula incorporates the primary taste cortex and together with the amygdala, which predominantly responds to emotions but also receives gustatory and other multimodal sensory inputs, form part of a brain network that regulates stimulus-response association and motivation to rewards such as food.

By suppressing postprandial release of gut hormones such as GLP-1 and PYY, octreotide administration has an advantage over specific antagonists in attenuating the additive effects of several satiety gut hormones to reduce reward-related eating behavior (17). A novel finding in both our studies was that octreotide reduced elevated postprandial plasma FGF19 in patients after RYGB. This could be via a direct effect on somatostatin receptors on enterocytes or perhaps mediated indirectly through changes in other gut hormone pathways. FGF19 has a central anorexigenic action in rodents (33) in addition to roles in regulation of liver, lipid, and glucose metabolism (34). Indeed, weight loss after vertical sleeve gastrectomy in rodents is dependent on intact signaling at the farsenoid-X receptor that mediates the stimulation of FGF19 secretion by enterocytes in response to bile acids (13). In our fMRI study, although increased reward system BOLD signal with octreotide correlated with the suppression of PYY in the combined bariatric surgical groups, with a similar trend for GLP-1, this was not seen for FGF19, suggesting a more prominent role for PYY/GLP-1 in mediating these changes. Nevertheless, a potential role for FGF19 in reward-related eating behavior would be worthy of further study.

Whereas octreotide-induced hypoinsulinemia could contribute to the increased breakpoint in the PRT study and enhanced food reward (35–37), increases in hedonic-brain responses to food in the fMRI study were still seen despite its prevention by coadministration of exogenous insulin. However, this is a potential limitation in the interpretation of the PRT study. Furthermore, the relative hyperglycemia seen after octreotide in both studies would, if anything, be expected to attenuate rather than enhance such reward responses (38, 39).

Activation of brain somatostatin 2 receptors stimulates feeding in rodents, raising the possibility of a direct brain effect of octreotide (40). However, the increase in PRT breakpoint for the sweet chocolate reward with octreotide in the RYGB group was not seen in the control nonobese group, suggesting a lack of nonspecific or direct central effects of the somatostatin analog. Similarly, the increase in food appeal and reward system BOLD signal with octreotide in the RYGB group was not seen in the matched BAND group. However, no significant difference was seen between the response to octreotide in direct comparisons between the RYGB and BAND groups. This is likely related to the small number of subjects studied and stringent statistics used but also due to the fact that octreotide does still have an effect to reduce postprandial PYY and GLP-1 secretion in patients after BAND. The attenuation of these satiety gut hormones with octreotide was lower in the BAND than RYGB groups, related to higher postprandial concentrations after RYGB. Together with the correlation results in the combined surgical groups, these findings suggest that gut hormones may have a concentration-dependent attenuating effect on the reward system BOLD signal after bariatric surgery.

A limitation of our study is the small number and power, especially in the exploratory fMRI study, meaning that the findings are in need of confirmation in larger studies. This may also have resulted in false-positive as well as false-negative errors, for example, in the analysis of the interaction with food energy density and lack of significance in the BAND group in the fMRI study. However, n = 9 in the BAND group had a power of 0.74–0.88 to detect similar differences in the BOLD signal with octreotide as seen in the RYGB group (see Supplemental Methods). Furthermore, we found a similar direction of effect of octreotide to increase food reward using three different measures (PRT breakpoint, food appeal, and BOLD signal in brain reward system during food evaluation) in two separate cohorts of patients after RYGB. We did not include an unoperated obese control group in either study, which would also have been of interest, particularly for the PRT study in which obesity may influence the breakpoint (7). However, the use of a BAND control group in the fMRI study, as an extension of our previous study (4), matched for BMI and weight loss, provided an ideal control for weight loss from a surgical intervention but without an exaggerated satiety gut hormone response. Additionally, our study investigated only the acute but not the chronic effects of satiety gut hormone suppression on food reward, and therefore, firm conclusions on their role in food intake and choice and long-term weight loss cannot be made.

In conclusion, acute suppression of the enhanced postprandial responses of satiety plasma gut hormones, such as PYY, GLP-1, and FGF19, increased both appetitive food reward in a PRT, and brain-hedonic responses to anticipatory food reward using fMRI, after RYGB. These results are consistent with the hypothesis that enhanced satiety gut hormone responses after RYGB, resulting from anatomical alteration of the upper gastrointestinal tract, contribute to the reduction in brain-hedonic responses to food after RYGB. This gives new insights into the increasingly recognized impact of gut hormones on reward-based eating behavior.
